# Resource Disambiguator for the Web: Extracting Biomedical Resources and Their Citations from the Scientific Literature

**DOI:** 10.1371/journal.pone.0146300

**Published:** 2016-01-05

**Authors:** Ibrahim Burak Ozyurt, Jeffrey S. Grethe, Maryann E. Martone, Anita E. Bandrowski

**Affiliations:** CRBS, UCSD, La Jolla, CA, United States of America; Swiss Institute of Bioinformatics, SWITZERLAND

## Abstract

The NIF Registry developed and maintained by the Neuroscience Information Framework is a cooperative project aimed at cataloging research resources, e.g., software tools, databases and tissue banks, funded largely by governments and available as tools to research scientists. Although originally conceived for neuroscience, the NIF Registry has over the years broadened in the scope to include research resources of general relevance to biomedical research. The current number of research resources listed by the Registry numbers over 13K. The broadening in scope to biomedical science led us to re-christen the NIF Registry platform as SciCrunch. The NIF/SciCrunch Registry has been cataloging the resource landscape since 2006; as such, it serves as a valuable dataset for tracking the breadth, fate and utilization of these resources. Our experience shows research resources like databases are dynamic objects, that can change location and scope over time. Although each record is entered manually and human-curated, the current size of the registry requires tools that can aid in curation efforts to keep content up to date, including when and where such resources are used. To address this challenge, we have developed an open source tool suite, collectively termed RDW: Resource Disambiguator for the (Web). RDW is designed to help in the upkeep and curation of the registry as well as in enhancing the content of the registry by automated extraction of resource candidates from the literature. The RDW toolkit includes a URL extractor from papers, resource candidate screen, resource URL change tracker, resource content change tracker. Curators access these tools via a web based user interface. Several strategies are used to optimize these tools, including supervised and unsupervised learning algorithms as well as statistical text analysis. The complete tool suite is used to enhance and maintain the resource registry as well as track the usage of individual resources through an innovative literature citation index honed for research resources. Here we present an overview of the Registry and show how the RDW tools are used in curation and usage tracking.

## Introduction

Scientific research resources such as databases serve an increasingly important role in science. However, the total number of scientifically relevant research resources and their use within biomedicine is not well understood. Methods for tracking research resources are not as well developed as those for cataloguing and tracking the biomedical literature, rather the web is full of partial lists maintained by various organizations. For example, the National Library of Medicine maintains 65 databases on its’ servers (http://www.ncbi.nlm.nih.gov/guide/all/, accessed January 20, 2015) including PubMed, PMC and NCBI Taxonomy, however they do not contain an authoritative list of all research tools available to scientists. Each institute maintains a list of resources that it has funded, e.g., National Institute on Drug Abuse (NIDA) (http://www.drugabuse.gov/research-resources). However, these are rarely complete and often out of date. The Nucleic Acids Research (NAR) journal maintains a list of about 1000 biological databases (http://www.oxfordjournals.org/our_journals/nar/database/a/) most of which have been described in their annual database issue, while the libraries’ WorldCat lists 4299 websites that contain the keyword ‘database’ (https://www.worldcat.org/search?q = database#%2528x0%253Aweb%2Bx4%253Adigital%2529format, accessed Jan 20, 2015) many of which are indeed scientific databases, that also appear in the NAR and the National Library of Medicine.

The Neuroscience Information Framework, NIF [1, 2], a Blueprint for Neuroscience Research funded project, was created to support neuroscience research by improving the ability to find, access and utilize this new class of research resources, defined here as data, tools, materials and services. Towards this end, NIF has been surveying and cataloging the research resource landscape since 2006 [1, 2]. The NIF maintains a Resource Registry that describes individual research resources through high-level metadata and a growing data federation providing deep access to many hundreds of biomedical databases. Although started as a neuroscience project, the field of neuroscience does not easily lend itself to a strict definition of scope, as almost all of biomedicine is potentially of relevance to neuroscience. As both the NIF Registry and Data Federation (http://www.neuinfo.org/nif_components/mynif.shtm) include a significant number of general biomedical databases and as the NIF architecture itself is generic, we have rechristened the NIF platform, SciCrunch (http://scicrunch.com) and its registry and data federation, the SciCrunch Registry and Data Federation. The NIF portal continues to provide a neuroscience-centered view into these data resources. However, SciCrunch now supports several additional domain portals into these data resources including the dkNET portal supported by the National Institute of Diabetes, Digestive and Kidney diseases (NIDDK). Thus, throughout this paper, the term NIF and SciCrunch will be used interchangeably.

Because NIF and now, SciCrunch, have been cataloging and tracking research resources for over 8 years, we have witnessed firsthand the mercurial nature of digital resources like databases. Databases and software tools, unlike books or articles, constantly change as do their metadata descriptions. This dynamism stands in contrast to current practices for the literature, where standards and best practices exist to ensure the stability of article content and links. Once published, the metadata surrounding an article rarely changes, except in the case of errata or addenda. Stable article metadata, e.g., authors, title, date of publication, are integral to our current citation system for articles, which allows us to determine when a particular article is cited.

In contrast, as we have documented, the metadata surrounding research resources constantly changes. For example, many databases can have various caretakers at different times, can change their URL addresses, can shift their focus to include species that were not originally included, and even change their names. Databases disappear if the websites are no longer maintained. The mercurial nature of research resources makes it difficult to keep the research registry up to date. The changing nature of even simple metadata like name, and location also makes tracking the utilization of these research resources difficult. Over the past 8 years, we have made thousands of manual updates to our resource registry. Despite moving the registry to a Wiki-based platform to help crowd-source curation, the size of our registry and the dynamism of the resource landscape is rapidly outstripping the ability for human curators to keep the registry current.

To address the challenges of cataloging and tracking research resources, we have developed an open-source tool suite named the Resource Disambiguator for the Web (RDW). RDW is designed to assist curators in identifying new resources, determining whether or not a resource has moved or changed in content and to detect when a particular resource has been cited in the literature. It enhances and extends some of our previous semi-automated curation routines developed using Textpresso [3]. The pipelines developed previously include a scoring metric, which downloads website content and scores the content using the NIFSTD ontology providing curators with text files for resource candidates as well as lists of resources that have significantly different numbers of matching terms. The methods described herein improve significantly the first pass methodology in several important ways: 1) by employing machine learning approaches to improve detection and dealing with false positive data 2) through the development of a database backend and 3) a graphical user interface (GUI) allowing curators to interact with the RDW system as opposed to accessing files.

Detecting resources in biomedical literature is of increasing interest and several approaches to this end are reported in the literature [4–6]. OReFiL [4] crawls MEDLINE abstracts and BioMed Central open-access journal full papers for URLs of resources using regular expressions and heuristic rules. Bioinformatics Resource Inventory (BIRI) [5] uses NLP techniques and handcrafted regular expressions expressed as transition networks for resource naming, functionality detection and resource classification for resource inventory generation. BIRI is generated on 400 biomedical abstracts. BioNERDS [6] is a named entity recognition (NER) system for detecting database and software names in the literature using extraction rules generated by examining 30 full text bioinformatics articles.

In contrast to the knowledge rich rule based approaches [5, 6], our approach relies on machine learning techniques to learn the rules to extract resources implicitly from simple annotation tasks (good/bad decisions) and uses active learning to further minimize the annotation effort which is the main bottleneck in a large scale continuous resource mention extraction operation involving millions of full text papers. Like OReFiL [4], our system extracts URLs via regular expressions, but also uses NER to detect resource names and searches publisher web sites for resource mentions. It provides machine learning based post filtering of all three aproaches for resource mention detection to incorporate annotator input for high precision results.

### SciCrunch Registry Overview and Analysis

The NIF Registry (RRID:nlx_144509) provides the entry point of all research resources searched via NIF. A subset of the databases in the Registry are more deeply integrated via the NIF Data Federation, which provides deep query of the contents of these databases [2, 7, 8]. As described in the introduction, the Registry is a human curated, catalog of research resources, annotated with the NIFSTD ontologies [9, 10] and curated according to curation guidelines (http://confluence.crbs.ucsd.edu/display/NIF/Resources+and+Curation) developed by NIF.

**Fig 1:**
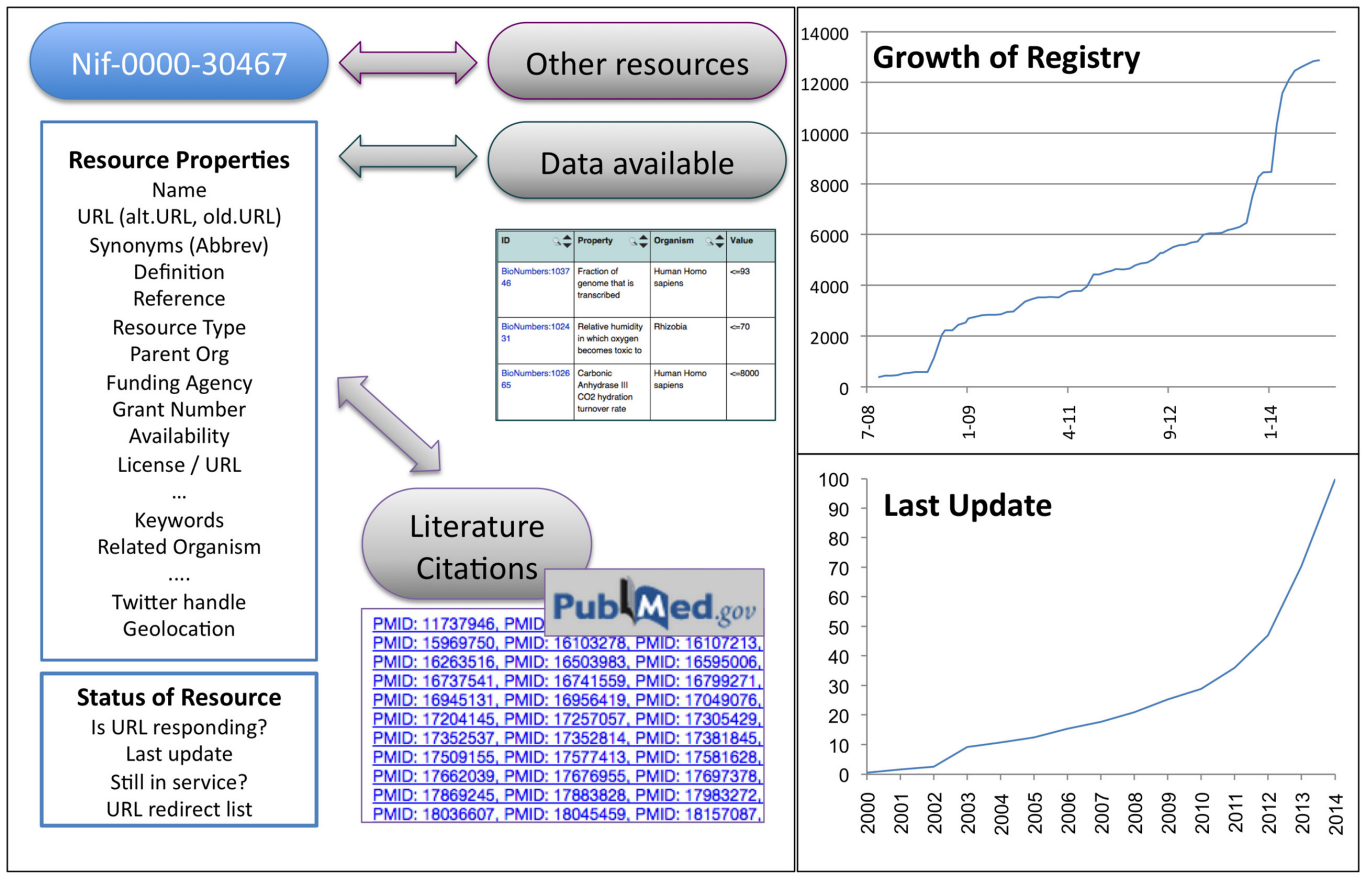
Overview of the resource registry. Left Panel: Figure depicts the registry entry for the ImageJ resource. Fields entered by curators include: Name, Keywords, Parent Organization, Supporting Agency, Reference, and URL / Alternative URL (not shown). The RDW generated fields include: Website Status and Mentioned in Literature aka the list of literature citations discussed in Section titled “Literature Management: Tracking resources mentioned in papers via automated tools’s web content over time / SPAM detector” of this paper. Top Right Panel: Figure depicts the growth of the registry over time, starting with 300 resources in 2008, and ending with 12,568 resources in 2014. Bottom Right Panel: The Figure shows the percentage of resources, that have a website updated date on the home page with a particular year. These dates were extracted in July 2014. The latest data from the NIF registry can be downloaded from https://www.neuinfo.org/mynif/search.php?t=registry. (Download of registry is available via a CSV file).

As described in our previous paper ([3]), each entry in the Registry includes a resource identifier, name, description, URL and resource type, which represents a set of metadata common across several major cataloging efforts in biomedicine including Biomedical Resource Ontology (BRO) [11] (RRID:nlx_143813), BioSiteMaps, and Eagle-i (RRID:nlx_144312) (See Fig 1). Indeed there are eight major types of resources: Data, Software, Training, Service, Material, Funding, People and Jobs. These eight major resource classes have 302 granular subclasses largely aligned to BRO and ERO and newer classes such as online course or mobile app that have not yet been reconciled. Most resources also include a subset of 30 additional attributes including parent organizations, supporting agencies, grant numbers, related diseases, access restrictions, related organisms, alternative or old URLs. All data in the registry is available not only as a searchable list, but also as a linked data graph. Each resource, is available as RDF served as part of the NeuroLex semantic media wiki. We include in S1 Text a copy of the registry, which is also available via a SPARQL end point (http://neurolex.org/wiki/NeuroLex_SPARQL_endpoint).

NIF also includes information on when the resource was last updated and if the resource has gone off-line. The graph in Fig 1 (lower right) shows the “last updated” data for the current contents of the NIF Registry. This information is extracted by curators and also through some of the automated pipelines described below. Of our current contents, relatively few resources have disappeared altogether (198 as of 10/2014). However, as shown in the graph in Fig 1(lower right), a significant number of resources are no longer actively maintained, as determined by their “last updated” or copyright date.

**Fig 2:**
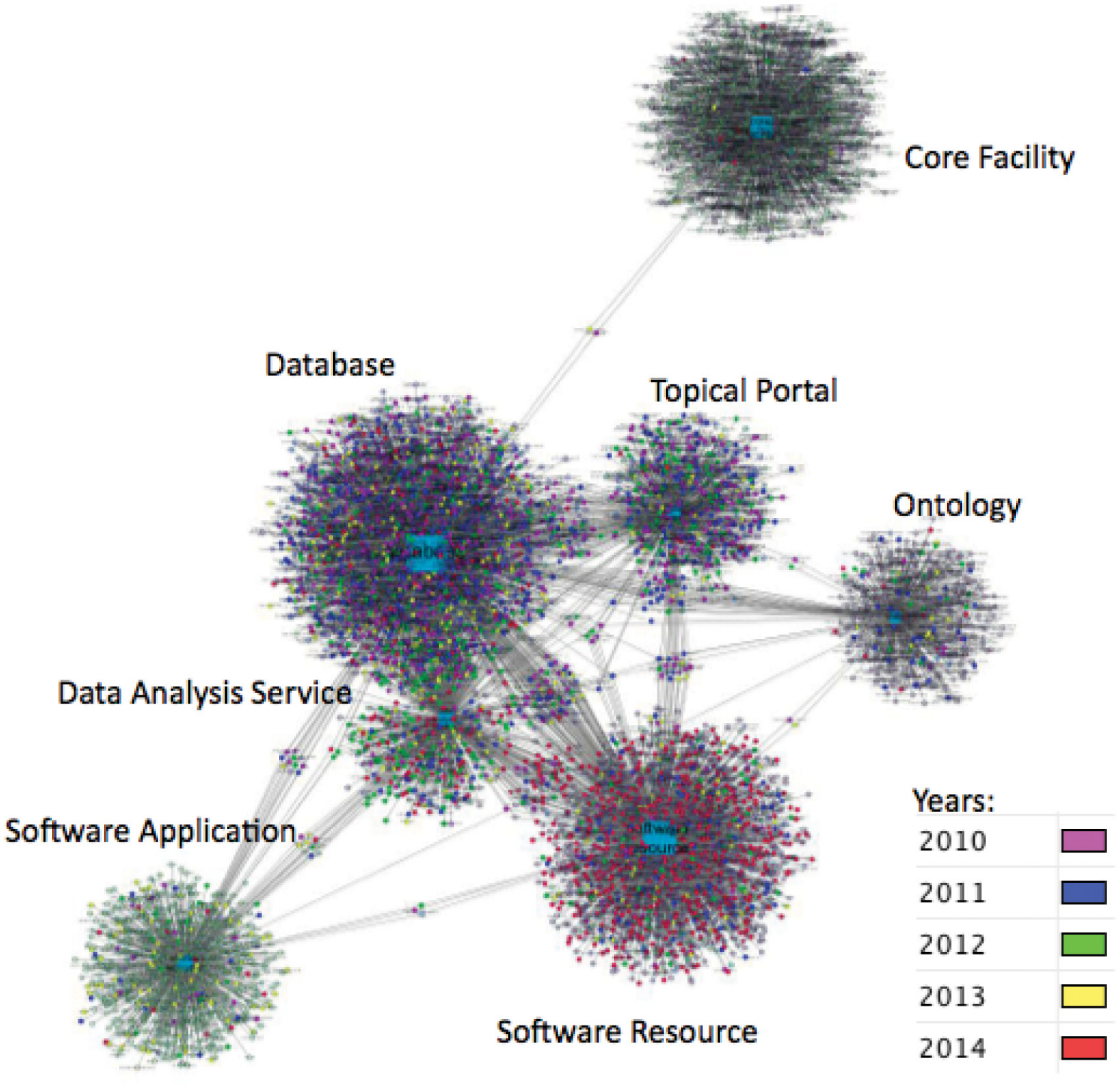
Resource types and year added to the registry. Research resources are each tagged with one or more resource types, the most common are represented in this graph (for all data see http://neurolex.org/wiki/Resource_Type_Hierarchy). The year that a resource was added to the registry is denoted by the color, note that 2009 and earlier data are lumped into 2010. Edges between points denote resources tagged with both, e.g. database and software. The SciCrunch registry data is available in S1 Text.

As shown in Fig 1 (upper right), the content of the NIF Registry has grown significantly since the initial launch of the pilot project in 2006 and first production release in 2008 [8]. An overview of the resource graph of the NIF Registry as of October 2014 is shown in Fig 2. Connections between resources represent resources that state a relation with another resource, e.g., the Protein Databank and the European Protein Databank. Resources are color coded according to the year they were entered. As can be seen, population of the NIF Registry was not uniform, with different types of resources being added at different times. This pattern largely reflects the ingestion into NIF of listings of research resources maintained by other organizations. For example, in 2013, a large number of software tools were added from the Genetic Analysis Software site (http://www.jurgott.org/linkage/ListSoftware.html). This comprehensive listing was maintained by a single individual that was largely neglected while the curator switched universities. The ingestion into the NIF enhanced coverage of genetic analysis software and served as a stable repository for this type of boutique collection.

## Materials and Methods

### Resource Disambiguator for the Web (RDW) System Architecture

The RDW (RRID:nlx_144525) tools were developed in order to provide curators with a set of automated and semi-automated tools to perform four major functions:

1. Determine whether the resource has moved
2. Ensure that the content is up to date
3. Track mentions of the resource within the biomedical literature.
4. Identify new resources from the literature: The creation of a new research resource is typically described in a publication that accompanies the release.

**Fig 3:**
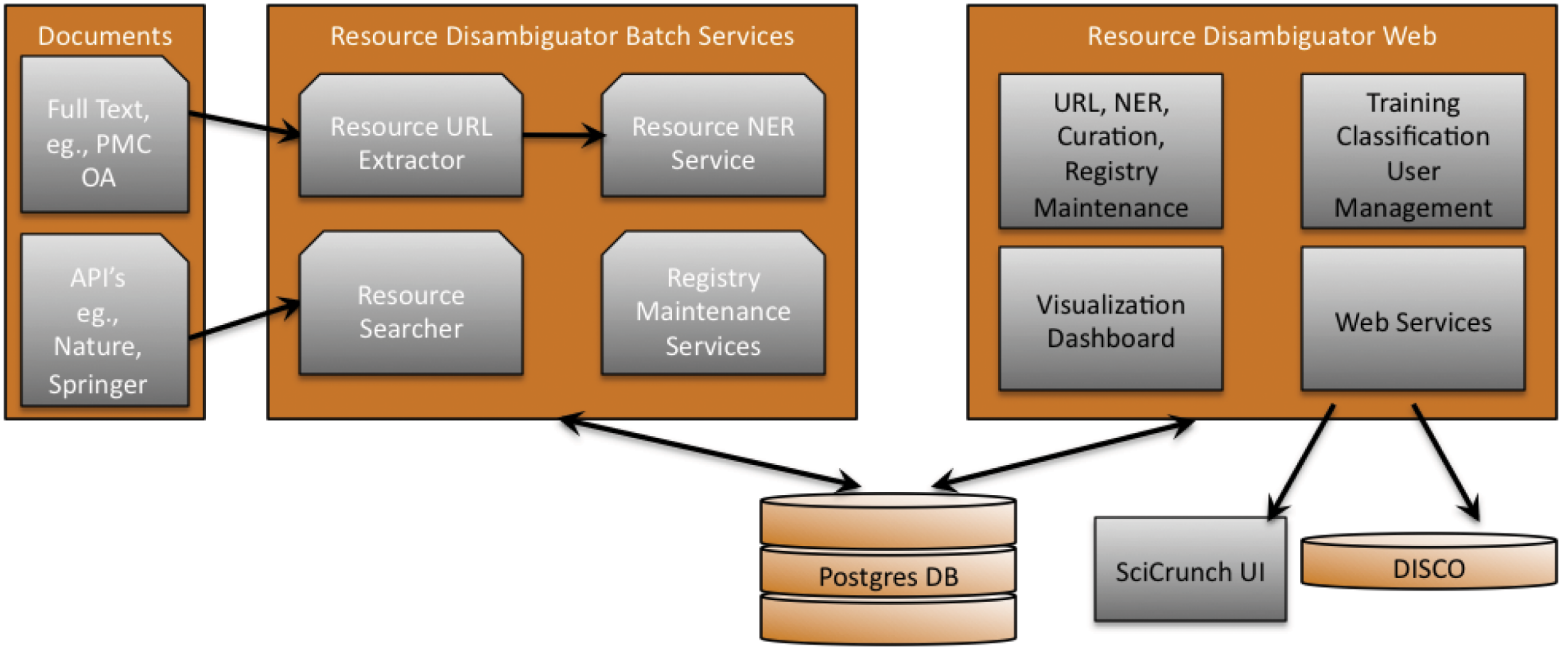
Resource Disambiguation System Architecture. The left panel represents literature sources including full text sources such as four Elsevier journals and the PubMed Central Open Access subset (∼1 million documents), and a set of services that grants access to PubMed abstracts (∼24 million documents), Springer and Nature content. The middle panel represents the set of automated tasks where data from the publications and the registry itself are processed. The right panel represents the set of curator facing dashboards that allow the curators to gain information from the batch services and give feedback to the algorithms represented in the middle panel. Finally, a Postgres database is the back-end data store and management system created to track the ongoing processes, compare and store all data. A set of web services is fed out that provides the output of pipelines and curation effort to the public. DISCO [7] is a data aggregation platform that feeds this data to the Neuroscience Information Framework project.

The web app code is available at http://dx.doi.org/10.5281/zenodo.20557 and the literature batch processing code at http://dx.doi.org/10.5281/zenodo.20560. Fig 3 shows an overview of the system architecture. Briefly, the Batch Services in the middle panel of Fig 3 represent a set of automated tools that extract likely resources from the literature, download the Registry, and run a set of tasks on each registry item. The Registry batch services include fetching the current registry data and a set of basic operations such as testing of the up/down status of registry URLs, testing the last updated date on the web site and the registry spam detector, which asks whether the content of a web site has significantly shifted. These services recapitulate and enhance the previously described curation pipeline developed using Textpresso [12].

The left side represents literature sources currently searched, which include the full set of abstracts from PubMed (RRID:nlx_82958, http://www.ncbi.nlm.nih.gov/pubmed), and full text sources such as the PubMed Central (RRID:nlx_18862, http://www.ncbi.nlm.nih.gov/pmc/) open access subset and a small number of journals from the Elsevier corpus. In total about one million full text articles are accessible. The full text literature sources are amenable to a thorough analysis of each sentence and were used to create algorithms that can detect resources in text. Unfortunately, the remainder of the literature remains somewhat opaque. Portions of the remaining 23 million biomedical research articles can be accessed via various programming interfaces such as PubMed (via NIF), Springer, or Nature application programming interfaces (APIs).

For full text papers, URLs and names of research resources (via named entity recognition, NER) are extracted from papers allowing us to ask about the resources used in the paper that may match an existing resource registry entry while non-matching mentions may represent a new candidate for the resource registry. The literature API batch operations are limited to asking each API for the set of names, synonyms and abbreviations for each research resource currently part of the Registry because, as mentioned above these APIs do not accept URLs as a query string.

The right side of the architecture diagram depicts the web interfaces where users interact with the batch operations and learning algorithms, including a dashboard overview of the performance of the entire system per resource. The interface is described in more detail in the next section. The system is underpinned by a Postgres database and interacts with NIF and SciCrunch public user interfaces via a set of REST web services accessed in most cases using the DISCO framework [7].

#### RDW Interface Overview

**Fig 4:**
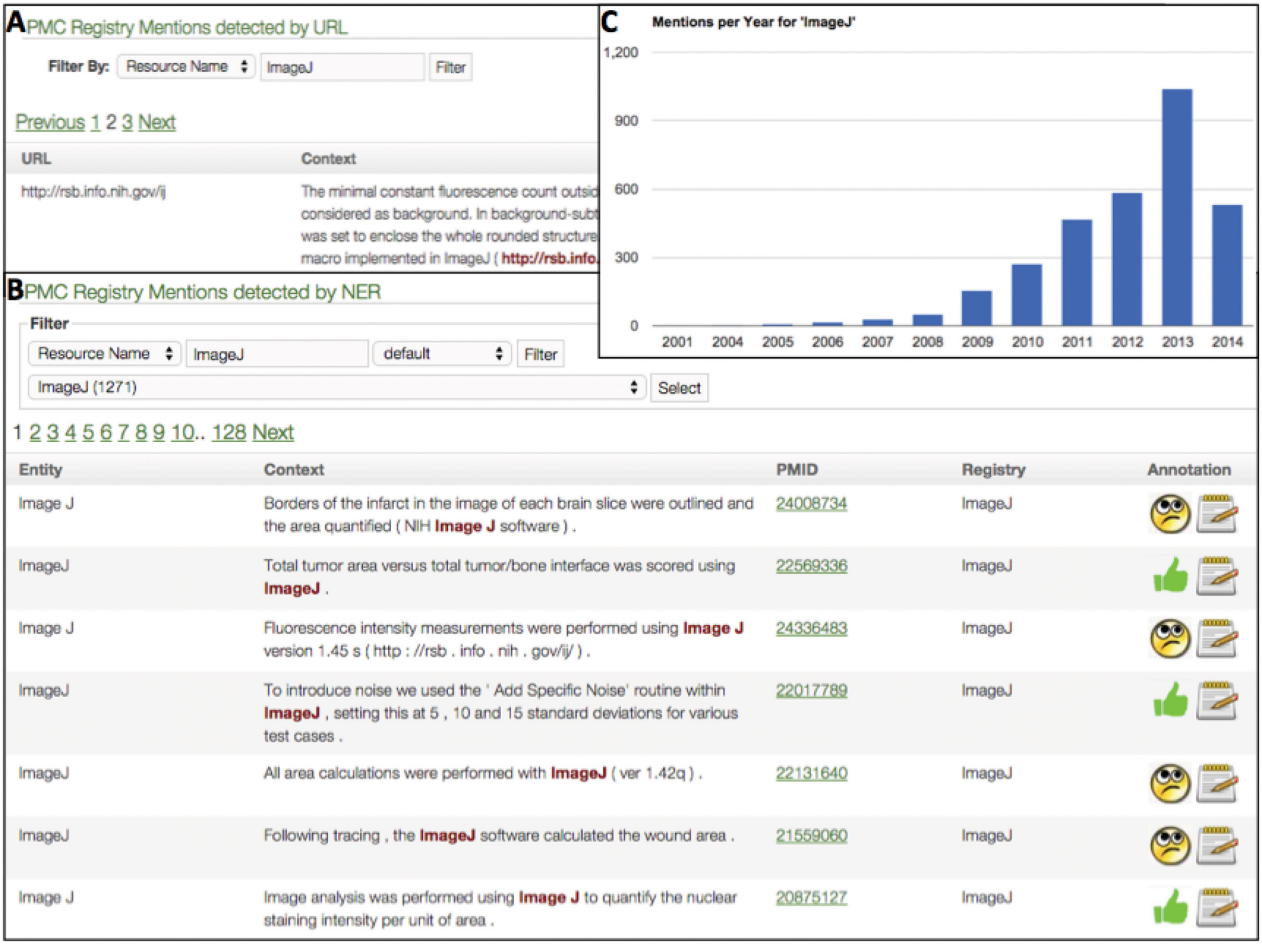
Screen shot of RDW user interface components. A. URL Mentions: Registry mentions interface component showing an exert of a paper containing the ImageJ URL. B. NER, Named Entity Recognition, Mentions: Registry mentions matches based on resource name, also for ImageJ. Curators toggled on the thumbs up icon for some of these mentions. C. Dashboard for individual resource: Graph of ImageJ resource mentions over time resulting from the all URL matches from A and curator annotations from B. ImageJ was first detected in a 2001 paper. Note that the same search in PubMed yields 2002 as the earliest mention. The main paper describing the tool is in 2004. Until 2008 there are only few papers detected for this tool.

The RDW web interface is built in GRAILS (https://grails.org/) and allows curators to interact with the system (Fig 4). Integral to the development of the learning algorithms is the ability for curators to provide input in the form of Good/Bad decisions for training each function. A good decision means that RDW performed correctly on a particular task while a bad decision means that it performed incorrectly. The curators render this decision by clicking on an icon (Fig 4B). In the example shown in Fig 4, which is asking the curator about a mention of ImageJ in the literature, a good decision means that ImageJ was correctly identified and a bad decision means that the resource identified was not ImageJ. The RDW system collects these decisions into the database. The interface also allows curators to visualize algorithm output and their decisions via several dashboards. As the curators are asked to provide feedback on several tasks, the interface is divided into several sections. However, in order to simplify the curator’s learning curve, all screens follow the same layout and require the same type of feedback.

The interface contains sections devoted to the following tasks. The details of the algorithms used in each task are provided in the next sections; here we provide an overview of each curator interface and any action required.

**Fig 5:**
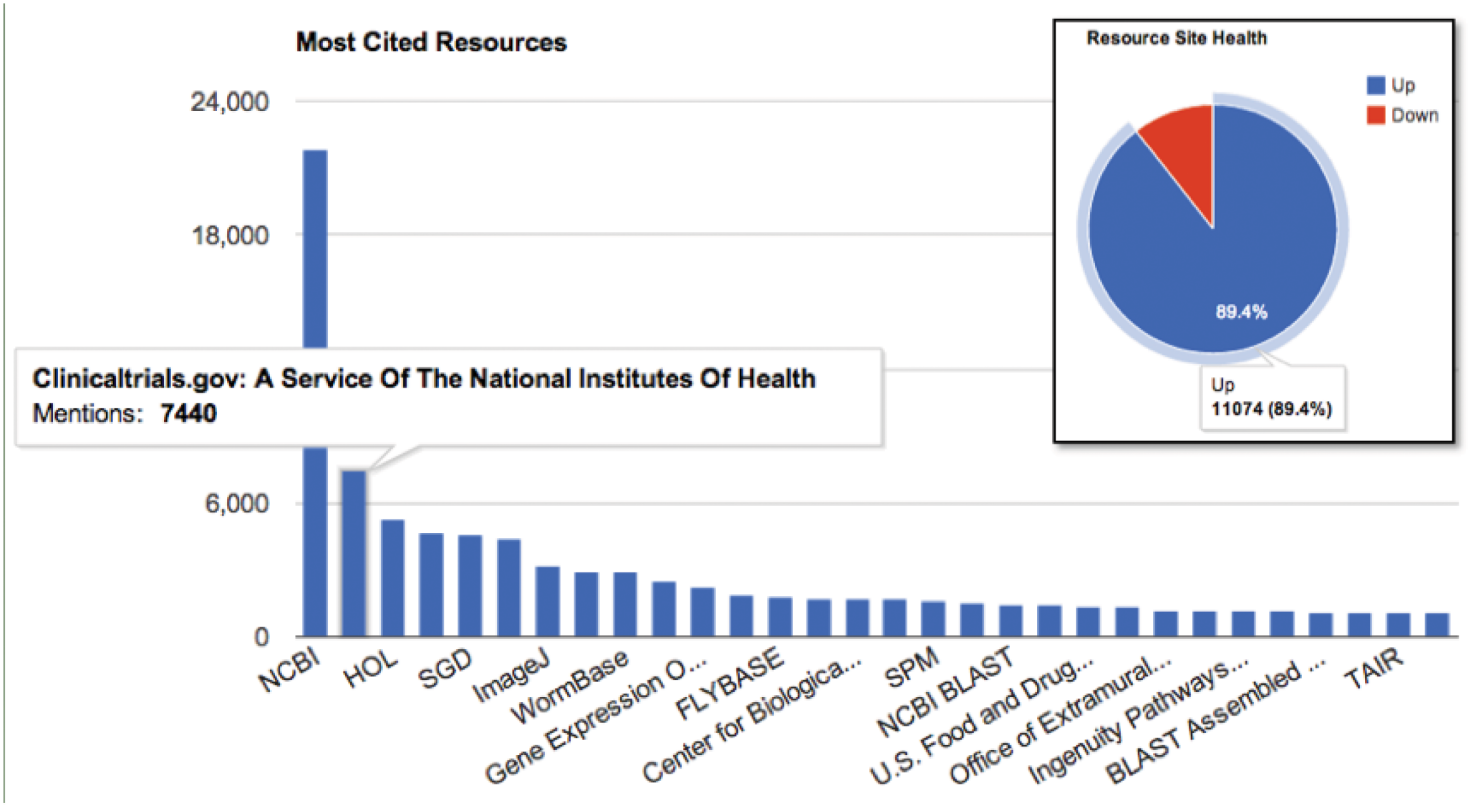
Analysis of URL mentions of research resources in papers. We show the top 30 resources sorted by the number of times URLs match a resource in a paper. Multiple mentions of a resource in a single paper are not counted as additional data points. For this analysis there were 100K of 500K detected URLs that matched one of the following: the exact URL present in the resource registry, an alternative or old URL noted in the resource registry, a URL that redirects to a resource registry item, or a simplification of the URL construct to the core resource, for example http://clinicaltrials.gov/NCI12345 would be counted as a reference to ClinicalTrials.gov. 500K URLs were detected in 700K papers (open access subset of PubMed Central, accessed 9/2014), the 400K that did not match registry items were not included in this analysis, but are included in other analysis of the NER system. Inset: URL status of main web page as assessed the week of 8/15/2014. An automated tool checks each web site in the registry to determine if the site is up or down each week and results are accumulated for curators. Curators are alerted when the site is down for 3 consecutive weeks.

1. Determining whether a URL is functional:—Good/Bad curator decisions are captured to determine whether the site is actually down (“good”) or if it is not down (“bad”). The latter case happens, for example, when a website does not allow automated crawlers to view its content. A “bad” decision by the curator removes the down flag from the resource and removes the resource form subsequent web crawls.
2. Determining whether a resource has moved:—Good/Bad curator decisions define whether the algorithm properly redirects from an old or existing URL to a new one. This information is stored in the database for other functions including URL detection, where URLs found in papers can be properly aggregated to a single resource, e.g.,during its existence, NIF moved from nif.nih.gov to neuinfo.org. In the Registry database, both of these URLs are registered to NIF.
3. Determining Registry updates and spam detection:—This interface provides a list of resource entries that show significant change in their content as determined by three similarity measures (Jaccard Coefficient, Semantic Similarity and Cosine Similarity). We have found that this function is very reliable in detecting websites that are no longer functional and have been taken over by spam. If a resource is determined to be spam, curators manually update the Registry entry.
4. Finding mentions of a resource in the literature by URL:—Good/Bad curator decisions mean that the algorithm detected a good or bad match with a registry resource. The interface allows curators to view, sort and filter resource mentions. For each detected resource mention the surrounding context and paper information is also provided to the curator (Fig 4A). Curators can provide additional information about why a match is bad through the “Notes” field.
5. Finding mentions of a resource in the literature by name:—Good/Bad curator decisions mean that the algorithm detected a good or bad match with a registry resource name, and synonyms stored as part of each registry record (for listing of all registry fields see Fig 1 left panel and S1 Text), see Fig 4B for an example match of RDW and ImageJ.
6. Finding mentions of a resource name via the literature APIs:—Good/Bad curator decisions mean that the API produced a good or bad match with a registry resource. The interface allows curators to view, sort and filter resource mentions, but as content is closed source curators must go to their library to find the paper by identifier and attempt to determine where the mention occurs.
7. Finding new resource candidates mentioned in the literature:—This screen (See S1 Fig) provides curators with likely resources as determined by text mining that do not currently match any Registry entry. The Good/Bad decisions are captured per Type of resource for the most common types including database, software tool and tissue bank. Curators are presented with the possible resource and the surrounding text for context. If curators find a suitable candidate they can create an entry in the Registry. If curators find that the URL is an alternate to an existing resource, a structured note is made and the URL is added to the Registry entry.
8. Visualizing results:—RDW contains three different visualization dashboards that provide an overview of current resources. They can display system status as it pertains to the most common tools detected in the literature via different routes, the resource mentions per year for individual resources (Fig 4C), the overall up/down statistics (Fig 5 Inset) as well as the co-occurrence of registry entities in papers (See S2 Fig).

RDW also has administrative functions for user management and management of training of classifiers from the curator provided good/bad decisions and classification of the identified resource mentions.

RDW provides REST web services for data harvest via DISCO [7], including the literature identifier for all high confidence resource mentions per registered resource and registered resource site up/down information. These data are displayed as part of the NIF / SciCrunch Registry user facing record where they are also available for download. High confidence resource mentions are defined as any mentions either directly made by curators or made by the RDW incorporated filter classifiers having a score (SVM classifier output value) greater or equal to 0.5 on the resource mention. The threshold is selected by changing the score threshold and checking a sample of the classifier selected mentions for errors. As one may note on Fig 4B, where the thumbs up is marked for some mentions of ImageJ, curators do not have time to test each new resource mention. However, we hypothesize that resource owners will be interested in all papers citing their resource and will be willing to provide good/bad information to train/refine RDW algorithms. To take advantage of this source of data, the registry will be moving to an extensible interface (SciCrunch) where several RDW tools will be exposed to resource owners not only curators.

In the following sections, we provide more detail on the algorithms used to perform the above functions.

### Registry Management: Tracking the up/down status of resources in the registry

For each resource in the registry, RDW checks the accessibility of the resource’s web site each week and keeps a history of the web site’s up/down status (See Fig 4). Because web sites can be intermittently down for maintenance or inaccessible due to temporary heavy load or network problems, a site is considered inactive only after three or more consecutive weeks of inaccessibility. Because a few sites reject access to non-browser based, automatic web agents there is possibility of false positives (tagging a resource as down even though it was up). To remedy this, RDW has a screen for curators to check if the latest inactive resources (down for three or more weeks) automatically detected by RDW are valid or not and provide feedback to the system. The system will then exclude those false positives from the inactive resources list. RDW automatically adds the current up/down and inactive status to the Registry via web services; this information is displayed through the Registry in SciCrunch and NIF user communities. If a resource is determined to be dead, the tag “THIS RESOURCE IS NO LONGER IN SERVICE” is added prominently to the registry entry. We never remove a resource entry from the Registry, as these resources may have been referenced in various papers. Often times, our registry landing page is the only record remaining of a discontinued resource.

For each resource URL, RDW also attempts to provide information on the latest updates to determine whether a resource is actively maintained and kept up to date. To do this, RDW extracts all available text from the “home” or “about” page and looks for dates using regular expressions. For example text such as “Copyright © 2003–2014” on the following software tool page (http://www.bioconductor.org/packages/2.13/bioc/html/CSSP.html) produces a result of 2014 as the date noted by the system. Data from this service are included in S1 Text and show that out of a total of 13,000 records, 1597 include the year that the web site was last updated. Of those, 516 were updated within the last year, but 78 resources report dates before 2004 suggesting that a significant subset of web sites are essentially dormant. We note, however, that this data is not complete because many web sites either lack dates or the dates are not accessible to our crawler.

### Registry Management: Tracking the movements of a resource

Over years, resources may move to new web locations usually, but not always, leaving redirect information on the original location to the new site. Curators track movements of resources manually by noting alternate URLs and old URLs in the Registry. As with dead links, when URLs no longer contain the scientific content and no redirect can be found then the tag “THIS RESOURCE IS NO LONGER IN SERVICE” is added. In this case, curators keep the URL in the ‘oldURL’ field for purposes of tracking the resource in the literature. The number of resources with the out of service tag is relatively small, 198 (about 1.5%), but the number of resources that move, as measured by the oldURLs, is 733, again beyond the number that can easily be managed through manual curation.

The RDW tracks the movement of resources in a semi-automated fashion. RDW downloads the text content of home and about pages, strips html tags, and uses text analysis on this content to determine if a web site has moved. Using the content of the main pages of registered resources, classifiers are trained to detect if the resource redirects to a different web site. The problem is cast as a binary text categorization problem where if the content indicates a redirection it is assigned the label ‘good’ and all the others in the training data set are assigned a ‘bad’ label. We generated our training data set by accessing, downloading and searching the text of each registry resource web site for the phrase ‘moved to’ resulting in 178 entries of which 42 were labeled as ‘good’ (See S2 Text).

We tested both supervised and semi-supervised approaches. For the supervised approach, we used a linear kernel SVM. As the semi-supervised approach, we used transductive SVM [13]. The semi-supervised learning approach tries to leverage co-occurrence patterns between the labeled training data and unlabeled data from the same distribution to improve generalization performance. As learning features, we used the length of the document in number of tokens and the existence/non-existence of unique words in the training set (bag of words representation). Unique words are defined as the lower cased unique terms in the training portion of the data set. Any new words introduced in the testing set are regarded as nonexistent during the classification. Since the bag of words approach ignores the local dependency of words in human languages and treats each word independent of other words in the context, we also tested short phrases of two contiguous tokens (bigrams) as features. Usually ‘site moved’ documents are relatively short, therefore we used the length of the document as a feature to take this into account.

### Registry Management: Tracking the change of a resource’s web content over time / SPAM detector

In addition to resources changing URLs we also need to detect changes in content. Wholesale changes in content usually indicate that the URL now points to a SPAM site. However more subtle changes to resources constantly occur, for example updates of grant information, or even the scope and nature of a resource. In the Registry, curators keep a running log of changes and updates. Indeed the number of edits performed every year, except 2014, surpasses the total number of resources (see Table 1). Although these metrics are gross, i.e., they do not track whether the curator made substantial changes or just minor edits or whether the same resource was edited multiple times, they do tell us that each year every resource was potentially subject to some update. Given the number of resources and the rate of growth the ability of a human curator to examine each record is beginning to hit a ceiling. Automated tracking and detection of these subtle changes is important if the Registry is to be kept up to date.

**Table 1:** Curation edits made to Registry resources over time.

Year	No.Edits	Total Resources
2010	4195	3529
2011	7877	4640
2012	7741	5688
2013	9657	8461
2014	11154	13174

The RDW has compiled a set of algorithms to help curators prioritize resources for re-curation, such that their time is spent examining resources that have changed as opposed to those that did not. Therefore RDW downloads resource web pages, strips HTML tags, same as above, and compares the text over time. The comparison uses three different similarity measures to periodically check for changes on each registered resources web site. These measures are;

- Jaccard similarity and containment using w-shingling [14].
- Cosine similarity using TF-IDF weighted document vectors.
- Jensen-Shannon divergence between current and original Latent Dirichlet Allocation (LDA) topic distributions of the web content of the resource.

For Jaccard similarity, a shingle is defined as a contiguous subsequence of tokens in a document *D*[14]. W-shingling *S*(*D*, *w*) for a document *D* is defined as the set of all unique shingles of size *w* in that document. Jaccard index, a common metric for resemblance of sets, between two documents *A* and *B* can be defined in terms of w-shingling as follows

$$J\;{\left(A,B\right)}_{w}=\frac{\left|S\;(A,w)\cap S\;(B,w)\right|}{\left|S\;(A,w)\cup S\;(B,w)\right|}$$

Since web sites frequently add new content, the following containment index [14] is also calculated for the current and original content of each resource;

$$c\;{\left(A,B\right)}_{w}=\frac{\left|S\;(A,w)\cap S\;(B,w)\right|}{\left|S\;(A,w)\right|}$$

Since the containment index is not symmetric, i.e. *B* can contain *A* (addition of new content to the original) or *A* can contain *B* (removal of content from the original), the maximum of *c*(*A*, *B*)_*w*_ and *c*(*B*, *A*)_*w*_ is used as the containment index for each resource. A shingle size of five is used which is arbitrarily selected with the goal of a phrase of length 5 distinctive enough to detect movement within a document and the number of resulting shingles are not too much to preserve memory. The w-shingling approach allows for location invariance of phrases of size *w* such that reshuffling of content has less impact on the similarity of the content.

For cosine similarity, a standard information retrieval (IR) similarity measure for vector space representation of documents, of the current and original content of a resource’s web site after stripping away the web markup tags, we used augmented TF-IDF to prevent bias towards longer documents. For the terms *t* in the documents (*d* ∈ *D*) augmented TF-IDF scheme is defined as

$$\left(0.5+\frac{0.5\times f_{t,d}}{fm_{d}}\right)\times \log\frac{N}{\left|\{d\in D:t\in d\}\right|}$$ (1)

where *f*_*t*, *d*_ is the frequency of the term *t* in the document *d*, *fm*_*d*_ is the maximum frequency of any term in the document *d* and *N* is the total number of documents. The site content (current and original) is tokenized, lower-cased, stripped of stop words before TF-IDF calculations. Cosine similarity between an original site content document vector (*x*_*o*_) and its current version (*x*_*c*_) is defined as

$$\frac{x_{o}\cdot x_{c}}{\sqrt{\left|\right|x_{o}\left|||\right|x_{c}\left|\right|}}$$

Since TF-IDF de-emphasizes terms that are used throughout the document set and emphasizes terms unique to each document, the cosine similarity will focus more on salient content words than functional words.

Latent Dirichlet Allocation (LDA) [15] is a generative probabilistic model for discrete data such as text documents. LDA models a document collection as a finite mixture over an underlying set of topics [15]. Given a number of topics, LDA estimates the mixture proportions over latent topics of each document, where each topic is represented by a distribution over words also estimated simultaneously from the training set of documents using maximum likelihood. How much the current content of a resource differs from the original content can also be modeled as the difference between their topic distributions. The standard measure for the difference between two probability distributions *P* and *Q* is the Kullback-Leibler divergence

$$D_{KL}\left(P||Q\right)=\sum_{i}\ln\left(\frac{P(i)}{Q(i)}\right)P\left(i\right)$$

If the two distributions are same, the Kullback-Leibler divergence is zero. Since Kullback-Leibler is unbounded otherwise and non-symmetric, Jensen-Shannon divergence, defined as

$$D_{JS}=\frac{1}{2}\left[D_{KL}\left(P|\right|\frac{1}{2}\left(P+Q\right))D_{KL}\left(Q|\right|\frac{1}{2}\left(P+Q\right))\right]$$

can be used, which is bounded between 0 and 1 and symmetric. Thus the document similarity can be defined as 1 − *D*_*JS*_. Since the words making up a topic is estimated from the whole training set of documents, one can filter out certain topics from the topic distribution of a document thus only focusing on the topics of interest for semantic similarity of content changes over time.

This tool has not yet been tested as to which method is optimal for detecting changes in web content, so currently curators are presented results from all three and update the registry entries accordingly.

## Literature Management: Tracking resources mentioned in papers via automated tools

This section describes the analysis of the literature, via different sources including PubMed Central, to find references to resources. We assume that resources are either 1) already in the registry in which case we aggregate the publications that describe use of those resources, or they are 2) not in the registry and we need to find ways to sort candidate resources for inclusion into the registry.

### Literature Management: URL Extractor

**Fig 6:**
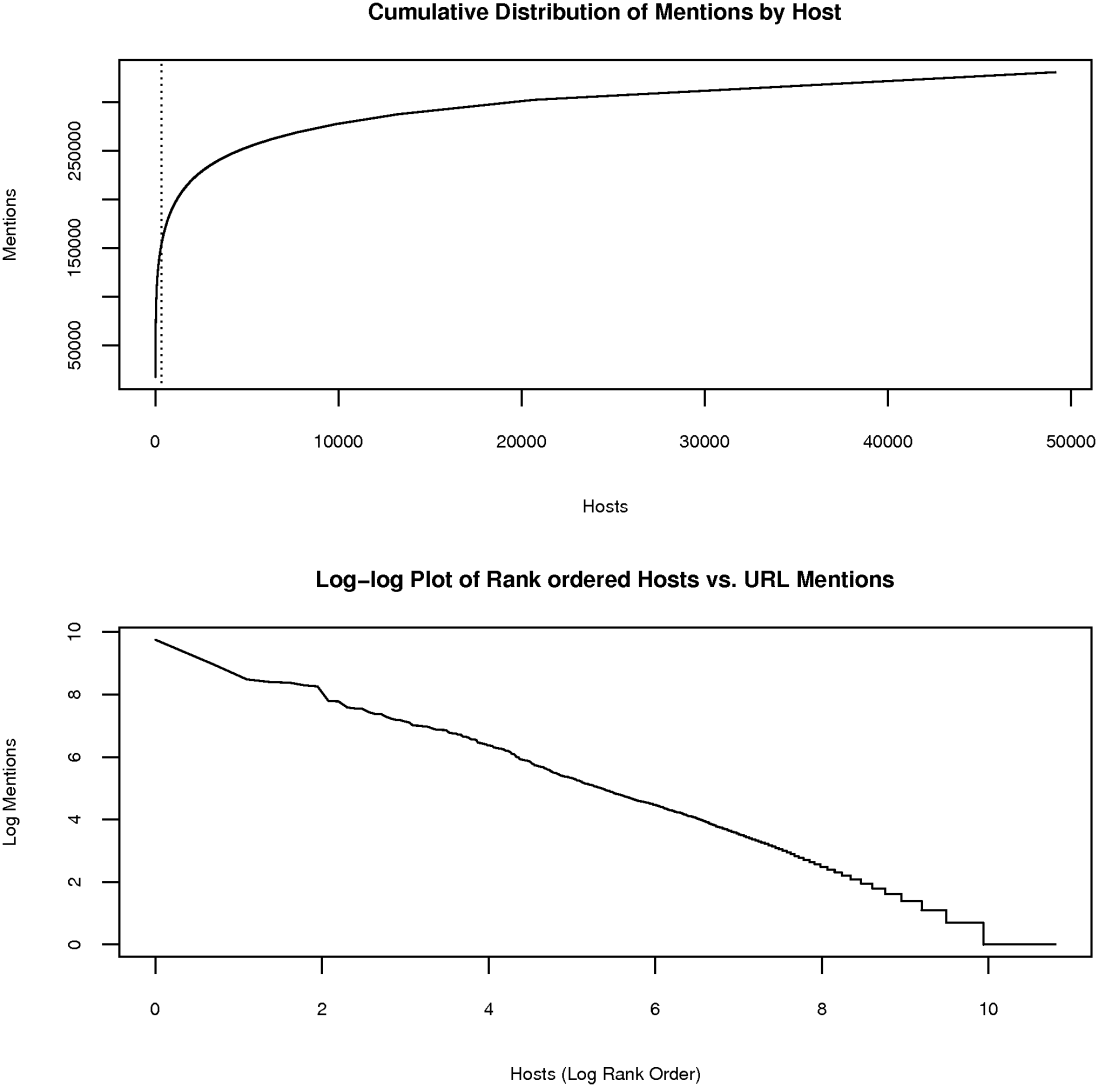
Cumulative distribution of mentions(y) by resource (host) (x). The dotted line separates hosts with 100 or more mentions (left side of the dotted line) from hosts less than 100 mentions. 327 hosts were responsible for half of the mentions. See S3 Text for a list of common hosts vs total number of papers. The near linear log-log plot of rank ordered hosts versus URL mentions indicates a Zipfian distribution.

Referencing a resource via URL is one of the major ways that research resources are mentioned in the literature [3]. PubMed Central open access journals are provided in the Journal Archiving and Interchange XML format with an explicit tag for URLs facilitating URL extraction. We excluded references sections of the papers while extracting URLs. To disambiguate URLs detected in full text papers, we first group them by URL host. An example of a host name is clinicaltrials.gov, in which case there are many individual trials that may be listed in papers such as https://clinicaltrials.gov/show/NCT00385866 and https://clinicaltrials.gov/show/NCT00335426. In most cases where individual data sets within a database are referenced, the host name represents the core research resource. The cumulative distribution of URL mentions in papers grouped by hosts is shown in Fig 6. As seen from this distribution the host versus URL frequency follows a Zipfian distribution, that is, a few of the hosts e.g. the Protein Data Bank and Wormbase, account for a significant portion of the mentions. (See Fig 5).

We use different approaches to analyze the cumulative URL mentions by host distribution from the exponential portion of the curve (many publications mentioning a resource) versus the long tail (few papers mentioning a resource). Resource candidates with 100+ mentions account for most of the unmatched URLs. Therefore, we chose a cutoff value of 100+ mentions. The 100+ mention resource candidates are aligned with resources via clustering and the remaining long tail is largely used to identify new resources using machine learning based filtering of new resource candidates as explained in the following sections.

#### Literature URL Extractor: Semi-automatic alignment of registered resources with extracted candidate web links

An extracted web link which does not match any of the SciCrunch registered resources may be one of several things: 1) a new resource; 2) an unknown alternate link for a known resource; 3) a service call to a service provided by a registered resource, for example a ftp site where data may be obtained. Due to the size of extracted mentions (more than a half million currently), a full inspection by humans is impossible. However, human inspection was used to tag the most common hosts (100+ mentions, for examples see Fig 4A). Therefore, a semi-automated approach was developed to align as many of the extracted unmatched web links exploiting the Zipfian form of the distribution of the web links grouped by host.

A large proportion of the extracted unmatched links can be accounted for by a few hundred of the largest host link clusters. To align an extracted link with a corresponding registered resource, a similarity measure needed to be defined. To this end, we used cosine similarity on document vectors of augmented TF-IDF (Eq 1) weighted terms. The automatic portion of the URL to the registered resource alignment by content similarity is detailed below;

- For each registered resource, using its web link, retrieve the web content and strip out the HTML tags (same as previous sections).
- for each registered site content, tokenize the content, lower case the terms, strip stop words and prepare term (*tf*_*i*_) and document frequencies (*df*_*i*_) for each unique term (*t*_*i*_|*i* ∈ 1…|*V*|). The vocabulary *V* is the set of all unique terms encountered in the registered resource home page set.
- Group extracted URLs by host and select the most popular hosts above a threshold (A threshold of a minimum of 100 mentions per host is selected). Each host is further grouped by unique URL path excluding any query parameters.
- For each selected host and unique path, retrieve the web content, strip the HTML tags, tokenize and lowercase the content, remove stop words and prepare term and document frequencies for all the unique terms occurring in the vocabulary *V*.
- For each host path cluster, prepare a TF-IDF weighted document vector (*x*_*c*_) and find the most similar registered resource as determined by minimum cosine angle between the cluster document vector and any of the registered resource document vectors (*y*_*r*_)
  $$\underset{r}{arg min}\frac{x_{c}\cdot y_{r}}{\sqrt{\left|\right|x_{c}\left|||\right|y_{r}\left|\right|}}$$

After this process, each host path cluster is assigned to its most similar registered resource. The automatic assignments can then be checked by a curator. Similarity values very close to one usually indicate a resource move and/or alternate URL. Similarity values close to zero usually indicate a potential new resource. Using this procedure, the number of web link mentions to be checked by the curators is reduced a few orders of magnitude from hundreds of thousands to a few hundred. Curators have gone through this set of data and added alternative URLs to existing resources and added new resources with over 100 mentions to the Registry. Although the total number of resources identified as high frequency cited resources is not a large number (about 300, with over 250 already in the registry prior to this process), the number of mentions in papers is significant. All results for matched resources are considered high-confidence, therefore included in the registry which track mentions of the resources in the published literature. These statistics are updated monthly with each new processed batch of papers. Resource mentions are available via services or in the Resource Registry interface (for example see Fig 1).

#### Literature URL Extractor: Resource Candidate Selection by Machine Learning for the resource mention distribution’s long tail

New unestablished and highly specialized resources form the long tail of the resource mention distribution. The tail of the distribution of the extracted web links shown in Fig 6 is very long and comprised primarily of new resources, since new resources will have very few mentions in the literature. Due to its sheer size, manual inspection of each extracted web link and its context is impossible. Hence, we cast the selection of actual resources from the list of extracted web links as a binary supervised text categorization problem. We filtered the tail of the web links distribution first by scoring them using NIF annotation service (http://nif-services.neuinfo.org/servicesv1/v1/annotate?content=<free-text>; where content is free text to be annotated with semantic types from NIF supported ontologies) as in Textpresso [12].

To generate this score, we retrieved the content of the extracted web link and annotated all the terms that match any ontology concept using the NIF annotation service. The resource eligibility score (Textpresso score) of the web link is the number of annotated terms from categories biological_process, anatomical_structure and resource in the content. We ranked the web link distribution tail by the Textpresso score and did not further consider any links with a score less than 10.

The filtered resource candidate web links are available to the curators through the RDW interface. The interface collects good/bad decisions from the curators to bootstrap and refine the text categorization.

Support vector machines (SVMs) are known to be highly suited for text categorization [16] where the number of features are very large but sparse. A bag of words representation is used for feature generation where each unique word is represented as a binary element of a document vector. The *SVM*^*light*^ package [16] is used for SVM training / classification. A linear kernel SVM with default *SVM*^*light*^ regularization parameter is used. The learning / classification cycles are managed by the RDW interface. The priming of the learning/classification cycles starts when the curators provide some reasonable (about 100 in this case) number of good/bad labels from the resource candidate links. After that, the first classifier is trained and used to classify the rest of the resource candidate links. In the following cycles either the supervised scheme is continued or active learning [17] can be used. In active learning, the classifier actively queries the oracle (curators) for the labels of candidate resource links for which the classifier is least confident. Active learning is usually used to minimize the number of label inquiries which translates into the minimizing of curator effort. For SVM based active learning, one way of selecting the candidate(s) for which the classifier is least confident is to select the candidates with minimum absolute classification score(s) i.e., the classification instances closest to the maximum margin decision boundary [17]. The RDW interface allows the curators to sort/filter the resource candidate links by either supervised or active learning scheme.

#### Literature URL Extractor: Experiments with interactive learning schemes for resource candidates

To evaluate the effectiveness of machine learning-based filtering of resource candidates, we used the resource candidates for the month of March 2014, filtered by Textpresso score of 10 or higher. This criterion yielded 870 unique resource candidate URLs used to generate a labeled data set. The first 601 out of the 870 resource candidate URLs, sorted by descending Textpresso scores, were annotated by a curator. Please see S4 Text for the annotated data set. The accuracy of the Textpresso score was 55.4%.

To simulate the interactive learning schemes introduced in this paper, we first randomly split the annotated dataset (601 resource candidates labeled either good or bad) in a stratified manner into a 70% training set and 30% test set. We generated ten such random splits. From each training set we selected one good and one bad labeled resource candidate as the seed set and assigned the rest of the training data as the pool from which to select training set candidates. Through the RDW interface, curators can provide feedback to the learning algorithm by providing a label either for the resource candidates for which the learning algorithm is most confident or least confident (active learning). The RDW interface allows ranking of the candidates both ways. For the active learning scheme, we first trained a linear kernel SVM on the seed data set. The classifier then classifies the resource candidates in the pool and selects the resource candidate with the least confident prediction to ask for a label. The selected resource candidate is added to the seed training set, removed from the pool and the classifier is retrained on the updated training set. This process is repeated 100 times resulting in a training set of 102. At each iteration the classifier is tested on the 30% test set. The most confident learning scheme experiment is similar to the active learning scheme experiment however instead of the least confident prediction, the most confident (maximum score) prediction gets a label and is included in the training set at every iteration.

### Literature Named Entity Recognition (NER): Resource Identification by Named Entities

In addition to searching for URLs, we also employ Named Entity Recognition (NER) to recognize resource mentions in the text by name. Although for some fairly unique names, e.g. ImageJ, Freesurfer, entity recognition is fairly straightforward, for others, particularly as acronyms are fairly common, challenges arise. For example Gemma is a common first name and a resource (http://www.chibi.ubc.ca/Gemma). So matches by simple lookup without taking account of the context in which the name is mentioned are poor. However, identification of resource mentions in biomedical texts can be cast as a sequence learning task. The Conditional random fields (CRF) [18] framework is one of the most popular probabilistic sequence learning approaches and has been applied to many named entity recognition tasks including tagging biological entities such as genes, proteins, cell lines [19, 20]. Like these, we detect names of resources in text and describe the pipeline below for detecting existing resource mentions.

The CRF is provided with sentences where each token is labeled to demarcate the named entities of interest for training. Once trained, the CRF model will provide labels for each token of sentences extracted from biomedical texts. A label on a token indicates if the token is a part of a named entity (e.g. research resource). For labeling, the IOB format is commonly used. Below is an example sentence with a resource mention; The|O:{DT} same|O:{JJ} method|{NN} was|O:{VBD} used|O:{VBN} in|O:{IN} Delorme|O:{NNP} et|O:{CC} al.|O:{NNP} (|O:{(} 2007|O:{CD})|O:{)} and|O:{CC} is|O:{VBZ} implemented|O:{VBN} in|O:{IN} the|O:{DT} EEGLAB|B-RESOURCE:{NNP} software|O:{NN}.|O:{.}

In IOB format the prefix B denotes the beginning of a named entity. The rest of terms in the NE are denoted by the prefix I and terms not belonging to any named entity by O. We extended the IOB format to encode additional information, namely, part-of-speech tags delimited by curly brackets for each token.

#### Training Features

The features used for NER are summarized in Table 2 accompanied by example features for the word ‘EEGLAB’ from the above example sentence. The majority of the features used are binary orthographic features commonly seen in other NER systems and approaches. These features include the identity of the word at index *t* in the sentence, if a word is all in uppercase or not or prefixes/suffixes of the word of length two through four letters. Word class is first introduced in ABNER [19]. It replaces contiguous upper case letters with an ‘A’, contiguous lower case letters with an ‘a’, contiguous numbers with a ‘0’ and rest of the symbols with a ‘x’ in a word. Part-of-speech tag of each word in the sentence is the only syntactic feature used. The only domain specific feature is membership of a word/phrase to the resource names, acronyms and NIF-ID lookup tables generated from the NIF/SciCrunch registry. The same set of features are extracted also for the word before and after the current word in the sentence taking surrounding context into account.

**Table 2:** Features used for Resource Identification.

Feature Name	Description	Example (EEGLAB)
INITCAPS	Starts with a capitalized letter	Yes
INITCAPSALPHA	All letter word starting with a capitalized letter	Yes
ALLCAPS	All characters upper cased	Yes
MIXCAPS	mixture of upper and lower case letters	No
HASDIGIT	At least one character is a digit	No
NATURALNUMBER	Word is a natural number	No
REALNUMBER	Word is a decimal number	No
HASDASH	Has at least one internal dash	No
INITDASH	Starts with a dash	No
ENDDASH	Ends with a dash	No
SURROUNDPAREN	Surrounded by parentheses	No
PREFIX_*i*	Starts with the prefix of length *i* ∈ {2, 3, 4}	EE,EEG, EEGL
SUFFIX_*i*	Ends with the suffix of length *i* ∈ {2, 3, 4}	AB, LAB, GLAB
PUNCTUATION	Word is a punctuation	No
WORD	Word itself	EEGLAB
WC	Word class	A
POS	Part-of-speech tag of the word	NNP
IS-RESOURCE	Match word/phrase in resource target list	Yes

#### Training

The NER system was trained on a relatively small data set of 307 resource named entities from a random selection of papers from four Elsevier neuroscience journals (Neuroimage, Brain Research, Neurobiology of Disease and Journal of Neuroscience Methods)). We received monthly batches of to be published papers and used the first six batches for the annotation set generation. As NIF is interested in determining the usage of resources in the literature, we restricted our analysis of full text to the methods section. To bootstrap the annotation process, we did a lookup for resource names and acronyms from the Registry on the extracted method sections. The results are then corrected by an annotator. NIF has been granted a license from Elsevier to access the full text of certain neuroscience journals in publisher’s XML format. The annotated data is provided as a supplementary XML file (See S5 Text).

The methods sections of the PMC open access papers are determined by applying regular expressions on section headings of the papers. For each paper, we segment the method section into individual sentences using a SVM based sentence segmenter we implemented based on the features described in [21]. The sentence segmenter is trained on biomedical domain data. After tokenization, we apply part-of-speech tagging using OpenNLP (https://opennlp.apache.org/). The token and part-of-speech tag information is provided to the NER tagger for each sentence of the method section. For CRF training we used the MALLET [22] library.

## Results

We evaluated the classification performance using standard information retrieval measures, *precision* *P*, *recall* *R* and harmonic mean of precision and recall *F*_1_ as defined below and used these measures throughout all experiments in this paper;

$$P=\frac{\text{\# of correctly recognized good labels}}{\text{\# of recognized good labels}}$$ (2)

$$R=\frac{\text{\# of correctly recognized good labels}}{\text{\# of true good labels}}$$ (3)

$$\begin{array}{c} F_{1}=\frac{2*P*R}{P+R} \end{array}$$ (4)

### Literature URL Extractor: Resource Candidate Selection by Machine Learning Results

**Fig 7:**
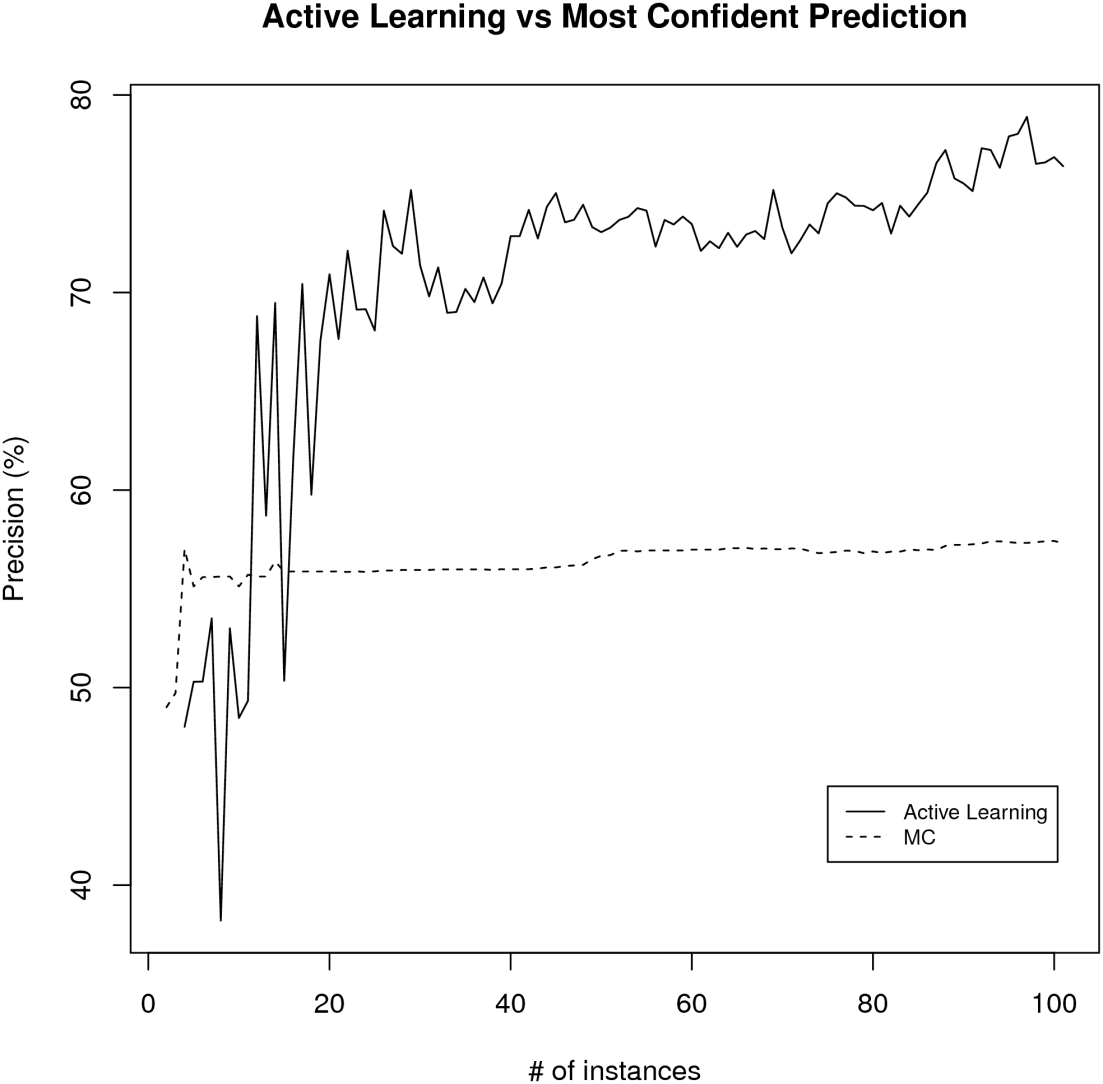
The average precision for active learning versus most confident (MC) prediction selection.

**Fig 8:**
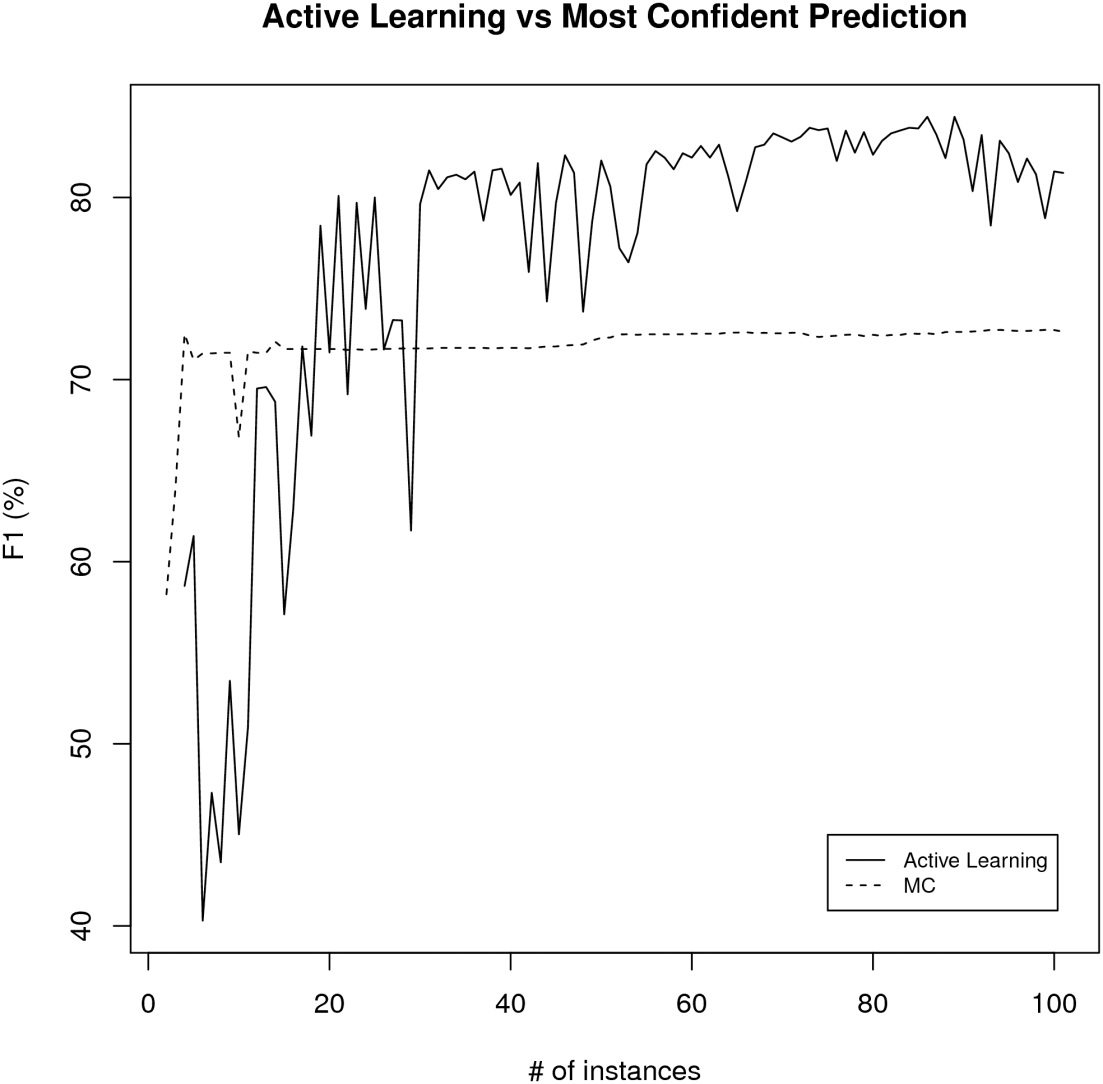
The average *F*_1_ for active learning versus most confident (MC) prediction selection.

The average precision and *F*_1_ curves for most confident learning scheme and active learning experiments is summarized in Figs 7 and 8. Based on the paired t-test on the area under the curve (AUC) values for both learning schemes over ten experiments, the active learning scheme is better than the most confident prediction selection scheme (p-value = 2.2e-6 for precision and p-value = 0.0013 for *F*_1_ case which are statistically significant). The average accuracy of the active learning based learning scheme after 40 iterations (42 curator labeled training set) was 77.6% with a standard deviation of 1.6% which has 22% higher accuracy than the Textpresso score reducing the error rate about 50%. The average is created from the ten training/test splits after 40 iterations of active learning. Therefore this is the method that is utilized in the RDW as the preferred method for finding resource candidates.

### Experiments for automatic detection of resource site movements

For training / classification of support vector machines, we used *SVM*^*light*^ package [16]. We generated ten 70%/30% random stratified splits and used them for our experiments. The average results for unigram/bigram features and supervised and semi-supervised (TSVM) machine learning approaches are shown in Table 3.

**Table 3:** Redirect Candidate Classification Results.

Classifier	Precision	Recall	F1
SVM (unigram)	80.57	80.83	80.17
SVM (bigram)	71.15	85.83	77.50
TSVM (unigram)	72.08	85.83	78.12
TSVM (bigram)	76.15	82.50	79.20

For supervised learning, bigram features seem to increase the recall at the expense of the precision. We conjecture that this is due to the introduced dependency between terms using bigrams more discriminative features can be represented, such as the bigram ‘moved to’ as opposed to the single terms ‘moved’ and ‘to’ occurring anywhere in the text.

Semi-supervised learning has good recall and somewhat lower precision than the supervised learning. The approach with the maximum *F*_1_ was incorporated into the RDW system to provide curators a list of resource redirect candidates. Furthermore, the URLs identified by the algorithm have been added to the registry and are tracked and used in all subsequent tasks (e.g. URL extraction).

### Resource Identification by Named Entity Recognition

We created ten training/test sets by randomly selecting 30% of the articles as the test set and the rest as the training set for the NER system. We trained ten NER systems on the 70% training data and tested each on their corresponding test set. On average the NER system had 89.2% (SD: 4.0%) precision and 88.8% (SD:4.2%) recall (*F*_1_: 89.0%, SD: 4.1%).

### Literature Resource Identification: Active Learning based Filtering of Springer, Nature and NIF Literature Searches for Resource Mentions

The above approaches for resource mentions were applied exclusively to the full text of articles from the PubMed Central. However, the vast majority of the literature could not be accessed by these methods. For the remainder of the literature we relied on two types of search APIs: PubMed abstracts via NIF Literature (http://nif-services.neuinfo.org/servicesv1/resource_LiteratureService.html#path__literature_search.html) and publisher-provided APIs that search full text from Nature (http://www.nature.com/developers/documentation/api-references/opensearch-api/) and Springer (https://dev.springer.com/). This set of APIs yield by far the largest resource mention candidates (close to two million resource mention candidates currently), since they access collectively close to 23 million articles. However due to their closed source nature, we have no control over the search algorithms and how much context (if any) around the search match is returned.

To test the accuracy of these results we selected a resource (ModelDB (http://senselab.med.yale.edu/ModelDB/)) and annotated each of the 443 results returned from the publisher search API queries as being a valid mention or not. The dataset is available as a supplementary document (See S6 Text). Out of 443 candidates, 179 were validated by curators as ModelDB mentions with an accuracy of 40%. We therefore consider these results as “low confidence” and expose them only to the curators until we can be certain that these are accurate. Because they are subject to many errors, we currently, do not include any of these matches in the registry output.

To simulate an active learning scheme for search result candidate filtering, we randomly split the annotated 443 resource mention candidates into 70%/30% training and testing sets, respectively. We repeated this procedure ten times to generate ten sets of 70%/30% random splits. Given one such random split, we randomly selected one good and one bad labeled resource mention candidate as the seed set to train a linear SVM classifier to start active learning iterations. The remainder of the training data is set aside as the pool set. As training features we used the title of the paper, the journal in which the paper is published, the description/context returned with the search result (if any) and MESH headings (for NIF Literature search results only). We combined the text from these search result fields into one document and using a bag of words representation generated a binary feature vector of occurrence of non-stop words for each training/test resource mention candidate. We also used the resource’s name as an additional feature to learn resource specific words for discrimination.

**Fig 9:**
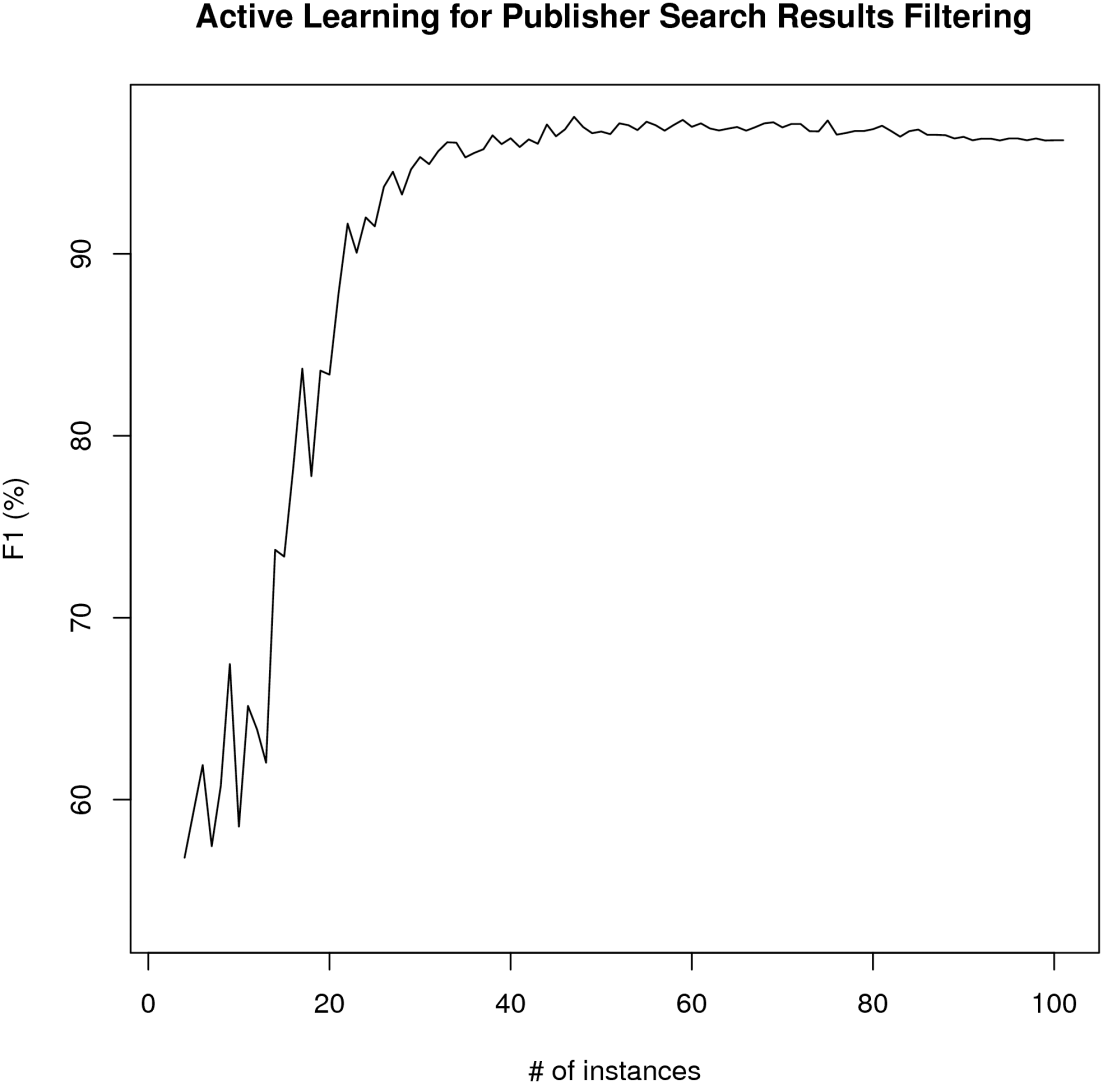
The average *F*_1_ for active learning to filter publisher search results.

At each iteration of the active learning scheme, we train the SVM classifier on the current seed set and use the trained classifier to classify the pool set and select the pool instance with the least confidence (the absolute minimum score) to add to the seed set and remove from the pool set. To see the classification performance at each active learning iteration, we classify the 30% test set using the SVM classifier trained on the current seed set. We repeated this procedure ten times on each random split. The average *F*_1_ curve for 100 active learning iterations is shown in Fig 9. The average accuracy of the active learning scheme after 30 iterations (32 curator labeled resource mention candidates) was 97.3% with standard deviation of 0.4% compared to 40% accuracy of search results before filtering.

Therefore, once sufficient curation feedback is given, the algorithm can detect with reasonable accuracy correct mentions of a resource even for this low confidence match set. All data with sufficient training is released as part of the SciCrunch registry comprising data for the literature citation field. However, it should be noted that training for one resource does not necessarily enhance accuracy across resources. We would therefore require training data from curators for each source, a time consuming task for curators. We are considering crowd-sourcing this task to the resource owners.

### Test of Coverage of RDW

**Fig 10:**
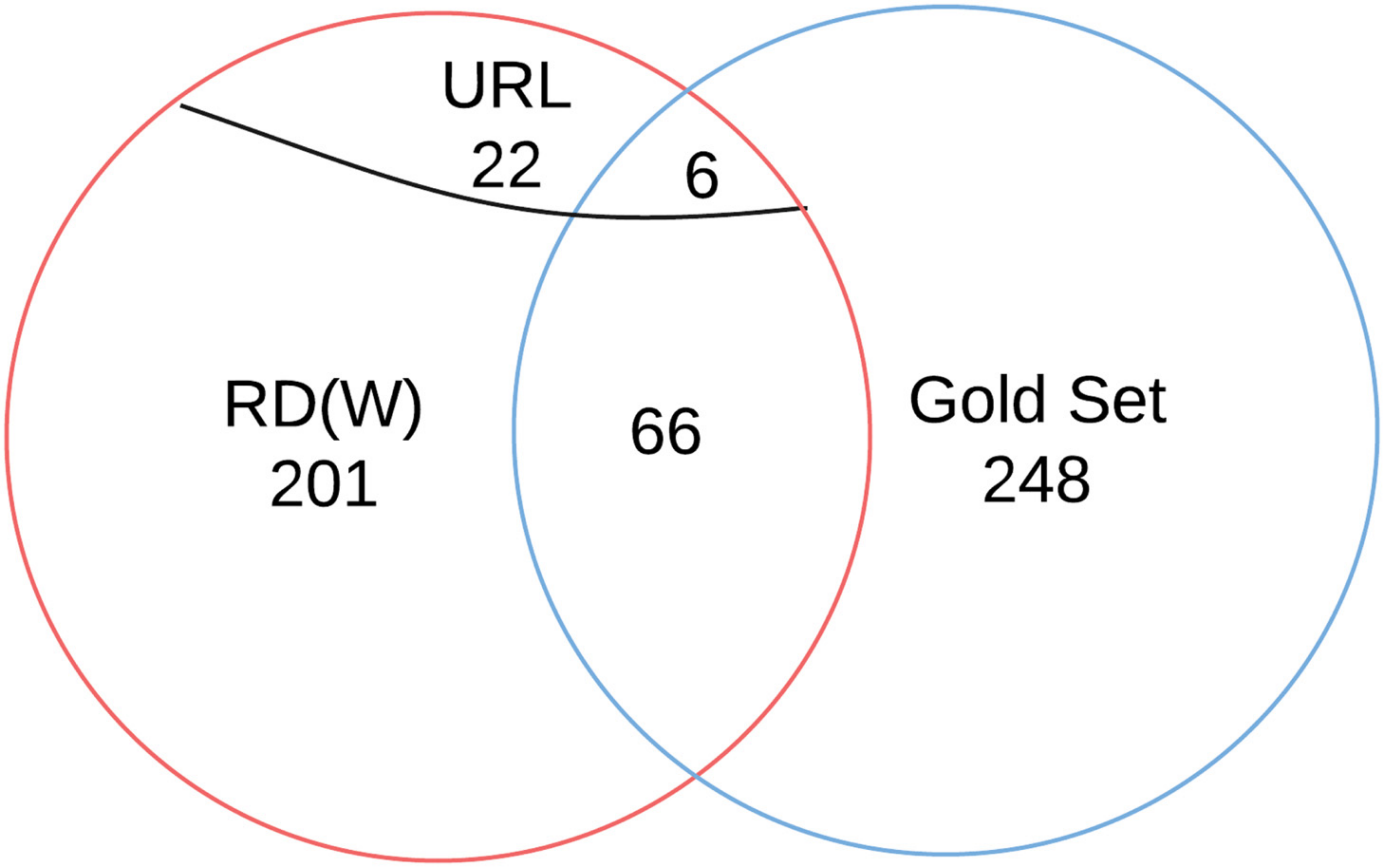
RDW literature coverage on ModelDB literature mentions test data set. The RDW pipeline results are shown along side of a Gold Set of manually annotated papers that mention ModelDB, downloaded from http://senselab.med.yale.edu/modeldb/ptrm.asp (June 2014). The RDW data set includes mentions by name and URL from journals that are the open access subset of PubMed Central, as well as Springer and Nature APIs. RDW found 201 valid papers that mention ModelDB, and 72 of those were in common with the Gold Set. Of the 201 mentions, URLs were detected in 22 cases, and of those 6 were in the common set. Please see S7 Text for the complete data set.

To test the coverage of our three approaches to identifying resource mentions, we used the list of manually annotated papers that mention ModelDB, downloaded from http://senselab.med.yale.edu/modeldb/ptrm.asp (June 2014) as the gold standard (248 entries with PubMed IDs). Fig 10 shows coverage of the three resource mention extraction approaches. The three approaches combined found 471 papers that mention ModelDB out of which 201 were valid, 72 of which were in common with the gold standard set. Of the 471 mentions, URLs were detected in 28 cases, six of which were in the common set. Therefore RDW found 22 high confidence mentions of ModelDB missed by the ModelDB staff. Out of the 443 search results (471–28 URL detections) ModelDB mention candidates, 113 were valid ModelDB mentions not present in the human curated data gold set indicative of increased coverage by the introduced method. These 113 new papers were of high interest to the principal investigators of ModelDB, who spend substantial effort pulling the information together from manual search efforts (personal communication T. Carnevale 2014). However, the system did not identify 176 papers that were in the gold set. We analyzed each missed ModelDB mention to identify the reason for the failure. The majority of the uncovered portion of the gold set (153 out of 176) was either in closed source journals (from other publishers such as Elsevier, Wiley, IEEE) or not included in PubMed Central open access data set (45 out of 151). Twelve misses were due to the Springer API not returning them in search results, another four were missing from Nature searches. To focus on resource usage, reference sections of papers are excluded from RDW URL mention extractions. Because of this, three mentions were missed. Another three were missed by NIF Literature search. Out of 176 uncovered mentions, 172 cannot be detected by RDW due to not having access to them. Out of the remaining four, one was missed by CRF NER and the other three by URL detector.

## Discussion

The NIF/SciCrunch Registry provides a uniquely valuable data set, both on the number and the state of digital resources largely funded through government investment and as a unique source of information on their utilization within the literature. To our knowledge, no other data set exists with the breadth of coverage and the rich metadata on the state of these resources over time. The Registry has both a large set of human annotated data, and, as described here, multiple machine learning pipelines all devoted to describing the state of research resources. The data set allows for an analysis of how many research resources there are in total, how frequently resources update and how many disappear or change university affiliations.

The NIF/SciCrunch Registry has grown every year, although we cannot claim that this growth reflects the actual production rate of new resources. As shown in Fig 2, the growth of the Registry was not uniform and reflected many factors, such as new types of resources for inclusion, e.g., Core facilities were added in bulk in 2014, and the incorporation of additional registries. Although we demonstrated the ability to detect new resource candidates from the literature, we find that determining the start date for a new resource is tricky. Digital resources may come on-line well before a paper about the resource is written, making information on the number of new resources produced each year somewhat difficult to ascertain.

As shown in the results, despite the fluidity of metadata surrounding these resources, the vast majority of them persist over many years, with less than 1% being completely decommissioned. However, the persistence of these resources does not mean that they are actively maintained, and we find evidence that a significant number grow stale, that is, they are still present but are no longer actively updated. For this reason, we now include the “last updated” date prominently in the Registry record through NIF and other SciCrunch interfaces.

As funders and researchers are starting to recognize research resources like databases and software tools as important products of scholarship, the ability to identify and track these entities is important. The pipelines described herein also provide the foundation for an analysis of the impact of research resources by tracking their utilization within the literature. Our analysis of citation patterns, indicates that the majority of citations of research resources are generated by a few well known resources, e.g., the PDB and NCBI/EBI supported resources. These results suggest that the majority of research resources are underutilized, although our analysis is clearly limited by the lack of access to the full biomedical literature. The inclusion of supporting information such as grant numbers and funding agencies within the Registry, combined with our text mining pipelines, should provide tools for assessing the impact of research funding. Although beyond the scope of this paper, we believe that the Registry data set and citation tools will also allow a deeper analysis of utilization patterns that may uncover interesting information on factors driving adoption, e.g., time in existence, number of updates, number of connected resources.

One of the major motivations for developing the automated pipeline described here is the daunting task encountered by curators in keeping the Registry content up-to-date and accurate. In this paper, we have demonstrated that semi-automated and automated machine learning approaches can be used to streamline curation tasks by identifying resources that have moved, disappeared or changed content. These same approaches were used to increase the efficiency and accuracy of our literature-based tools for tracking utilization of and identifying new research resources. Although we had previously reported on an automated resource identification tool developed using Textpresso [3], our experiments using machine learning indicate that active learning as a resource candidate filtering scheme allows better performance than the Textpresso-based methods and significantly outperforms most confidence-based resource candidate filtering at the same level of curation effort. The improvement in performance, particularly for some types of tasks, e.g., detection of site redirects (*F*_1_ 80%) and recognition of resource candidates from URLs, means that these routines are sufficiently robust that they can be incorporated into our curation pipeline.

The RDW tools described here have been in operation for over a year and are currently in use by the SciCrunch. They have detected more than 196,000 resource mentions in papers by URLs, 113,694 by named entity recognition and close to two million by publisher searches (as of 01/27/2015). All URL mentions (196,683) and 26,365 named entities with filter classification score greater or equal to 0.5 are considered high confidence matches and are made available to the public. Curators have added over 50 resources as a direct result of RDW identification.

The most successful algorithms and approaches were developed on top of access to the full text. Full text access is available for the PubMed Central Open Access subset and by custom agreement with certain publishers. Some publishers, e.g. Nature and Springer provide general APIs for searching their content. We found that just using these search-based APIs resulted in low quality results (∼40%) making this approach unusable for resource curation. The problems were mostly due to the word sense disambiguation problems leading to more false positives than other approaches. In addition, the search APIs are of limited functionality often returning insufficient context, making it practically impossible to verify if the match is an actual resource mention. However, we can, as shown with the example of ModelDB, train classifiers to significantly improve our ability to accurately recognize individual resource mentions, even within the closed access literature, but these classifiers are trained on a per resource basis, making scaling of this approach dependent on human curation. As having accurate statistics on how and when these resources are used is of great interest to individual resource providers, in the future we will enlist the resource providers to provide the training sets in this task through the development of appropriate interfaces.

The most serious limitation encountered was the lack of any full text access to the majority of the literature. We suspect therefore that we are missing a large number of resource mentions because we can only access the abstracts and many resources are likely mentioned only within the materials and methods.

## Conclusion

Biomedical science is increasingly a digital enterprise, involving the creation of digital assets such as databases, software tools and services. While significant government funding has gone to produce these assets, we as yet lack an infrastructure or set of procedures for keeping track of these assets and how they are used. The NIF Registry, renamed the SciCrunch Registry in recognition of its broadening coverage of biomedical science, is one of the first efforts to develop such an infrastructure. While many catalogs of research resources have been assembled, few have the breath or depth of data about these resources assembled over many years by the NIF. This data set gives a unique view into the state of digital resources, particularly their fluidity and dynamism relative to more traditional scholarly artifacts such as the literature. Because of this dynamism, where resources change in content, move locations and disappear, keeping the Registry current involves significant curation. As we have shown here, the size of this task makes the development and use of machine learning-based automated approaches critical. These routines work in tandem with the literature to identify new and existing resources. When our methods are applied to the full text literature, they are good enough to augment our Registry with little or no human intervention and provide useful statistics on resource use. However the majority of full text is not available to us, seriously hampering our ability to use automated methods. Thus, the main limitation in applying these routines wholesale is a policy and not simply a technical issue. The difficulties encountered in using abstracts and publisher APIs for resource identification argues for a push towards global machine-based access to the biomedical literature.

## Supporting Information

S1 Text Registry content. TAB separated file listing SciCrunch registry resources and curated attributes.
(CSV)

S2 Text Annotated data for redirect classification. An XML file listing resource redirection candidates annotated with good/bad labels used to train/test redirect detection classifiers. For each of 178 resource candidates the HTML stripped text of the about or home page, its registry NIF ID and url is provided along with its label.
(XML)

S3 Text Most mentioned new URLs as resource candidates. A text file listing new resource candidate URLs grouped by host with 100+ mentions in open access papers together with their closest aligned Registry resource and similarity score.
(TXT)

S4 Text Annotated data for resource candidate interactive learning scheme testing. An XML file listing resource candidates detected by the system in March 2014 labeled by an annotator as good or bad. For each resource candidate its Textpresso score, url and description is provided besides its label.
(XML)

S5 Text Annotated data set for resource NER. An XML file listing sentence fragments with their corresponding parse trees for the methods sections from four Elsevier journals (Neuroimage, Brain Research, Neurobiology of Disease and Journal of Neuroscience Methods) and resource name entities for them.
(XML)

S6 Text Annotated data set for ModelDB mentions from publisher and NIF searches. An XML file containing resource mentions detected by Springer, Nature, NIF search API searches for a resource’s name, synonyms and URL for the resource ModelDB annotated with a good/bad label used for active learning experiments. Besides the label each entry includes a title, publication name, description (if returned by the search API) and Mesh headings (only for NIF searches).
(XML)

S7 Text Data for Fig 10. Comma separated file listing ModelDB mentions found by RDW compared to human curated list of ModelDB mentions used to generate Fig 10.
(CSV)

S1 Fig Screenshot of the RDW resource candidates view. A page of curated resource candidates are shown.
(PNG)

S2 Fig Screenshot of the RDW resource co-occurrence heat map. The heat map shows the 50 most frequently co-occurring resource mentions in RDW database as of July 2015. The darker a cell the more are co-occurring mentions.
(PNG)
