# Supplementary figures and images for: Resource Disambiguator for the Web: Extracting Biomedical Resources and Their Citations from the Scientific Literature
Source: PLoS One. 2016 Jan 5;11(1):e0146300. doi: 10.1371/journal.pone.0146300 (PMC5156472; doi:10.1371/journal.pone.0146300)

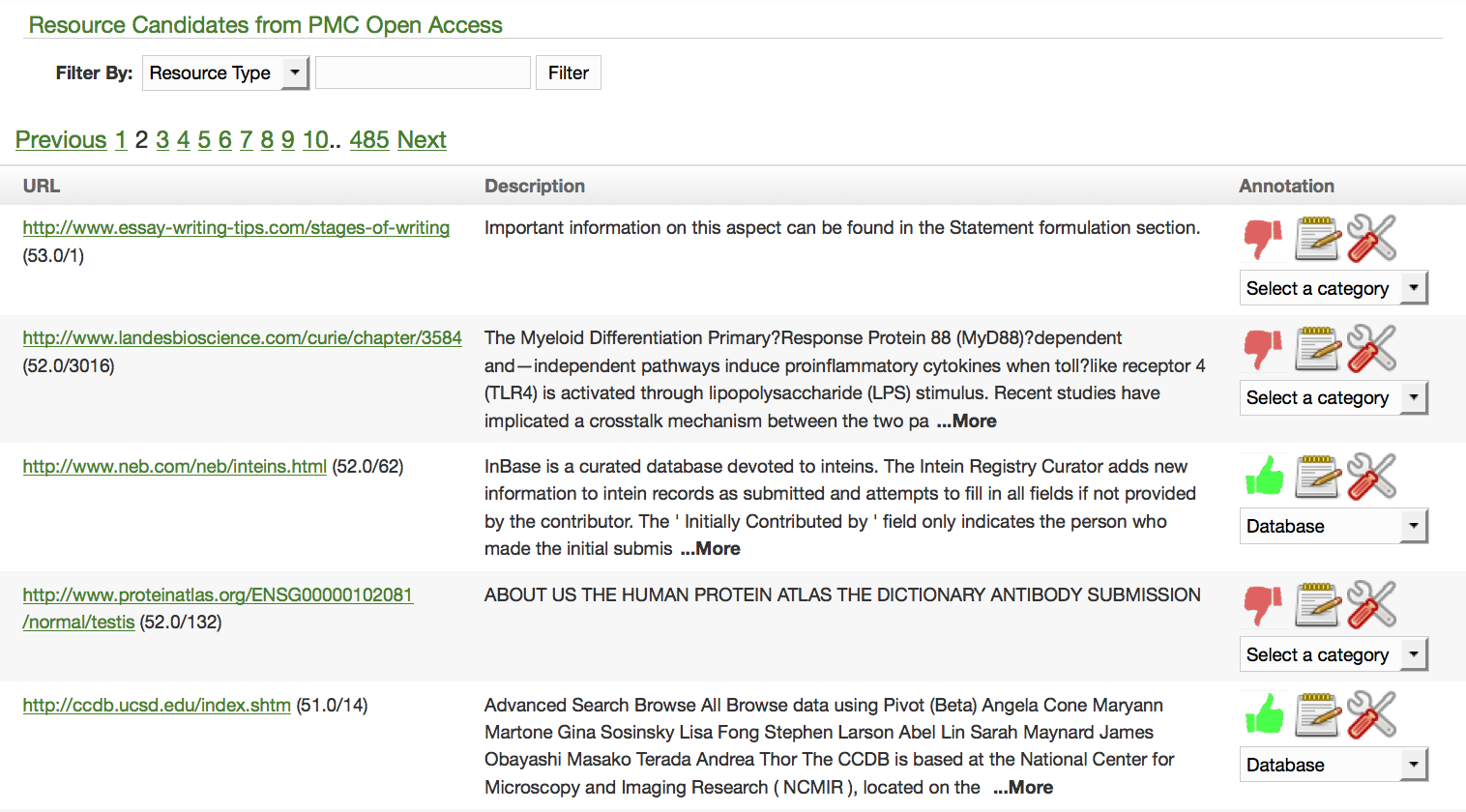

Supplement: S1 Fig — A page of curated resource candidates are shown. (PNG) [file pone.0146300.s008.png]

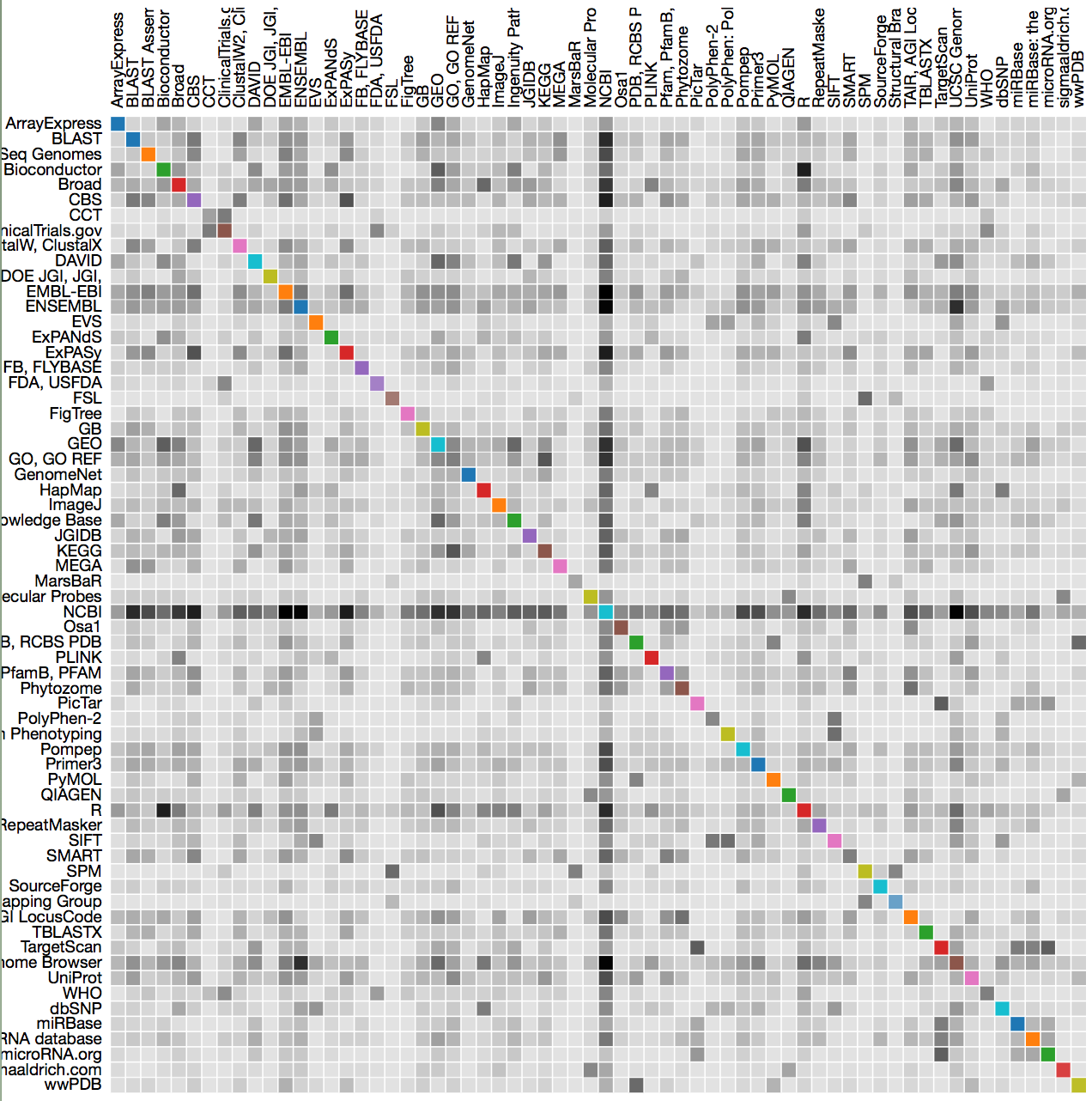

Supplement: S2 Fig — The heat map shows the 50 most frequently co-occurring resource mentions in RDW database as of July 2015. The darker a cell the more are co-occurring mentions. (PNG) [file pone.0146300.s009.png]
